# Cryorecovery of Mouse Sperm by Different IVF Methods Using MBCD and GSH

**DOI:** 10.4172/2375-4508.1000175

**Published:** 2016-03-18

**Authors:** Ming-Wen Li, Olivia C Glass, Jasmin Zarrabi, Lisa N. Baker, K. C. Kent Lloyd

**Affiliations:** Mouse Biology Program, University of California, Davis, CA 95618, United States

**Keywords:** Mouse, Sperm, Cryopreservation, IVF

## Abstract

Different protocols incorporating methyl-β-cyclodextrin (MBCD) and reduced glutathione (GSH) have been reported to improve IVF recovery of cryopreserved mouse sperm on a C57BL/6 (J and N) genetic background. However, it is not clear which IVF protocol is most appropriate when using the various methods to cryorecover sperm with different sperm quality and sample volumes. Therefore, in the present study we correlated sperm motility with fertilization rate and compared the efficiency of different IVF methods using various sperm samples so as to establish general guidelines for mouse sperm cryorecovery by IVF. High linear correlation between sperm fertilization rate and progressive motility was found, R^2^ was 0.9623 and 0.9993 for pre-freezing and post-thaw progressive motility, respectively. High amounts of cryoprotective agent (CPA) were observed to impair both sperm capacitation and fertilization. Moreover, the presence of a large number of immotile sperm in the sperm-oocyte co-incubation drop was found to reduce IVF success which could be partially reversed by supplementation using monothioglycerol (MTG) during centrifugation. It was concluded that the efficiency of IVF using cryorecovered mouse sperm in media containing MBCD and GSH can be predicted from sperm progressive motility. High concentrations of CPA and immotile sperm should be mitigated prior to IVF. The optimum IVF method should be selected based on sperm sample volume and sperm parameters.

## Introduction

Cryopreserving mouse sperm using 18% raffinose and 3% skim milk (R18S3) as a cryoprotective agent (CPA) has been widely used since 1990 to maintain genetically-altered mice in an inanimate state for archiving and distribution purposes [1,2]. Unfortunately, the reliability and efficiency of using frozen-thawed sperm for *in vitro* fertilization (IVF) is highly variable and dependent on genetic background, particularly for mutant mouse strains on an inbred C57BL/6 background [3,4]. The addition of monothioglycerol (MTG, an antioxidant) or glutamine (Glu) to R18S3 for sperm cryopreservation has been reported to significantly increase the fertilization rates of frozen-thawed mouse sperm [4–6]. Further, mouse sperm cryopreserved in R18S3+MTG has significantly better post-thaw progressive motility and fertilization rate than sperm cryopreserved in R18S3 alone or R18S3+Glu. Additionally, there has been no significant difference in fertilization rate reported between sperm cryopreserved in cryovials as compared to cryostraws [6].

Although the use of CPA containing MTG or Glu has increased the efficiency and utility of mouse sperm cryopreservation, fertilization rates of frozen-thawed sperm remains unacceptably low when using conventional IVF methods [6,7]. An IVF method using sperm pre-incubation in methyl-β-cyclodextrin (MBCD) to enhance sperm capacitation and enrich progressively motile sperm to the periphery of the MBCD drop and oocyte pre-incubation in IVF medium containing reduced glutathione (GSH) to modify zona pellucida structure for easier sperm-zona penetration (termed “MBCD-GSH IVF”) has been reported to significantly increase the fertilization rate of frozen-thawed wildtype and mutant mouse sperm on inbred C57BL/6 (J and N) backgrounds [5,7–9], in which mainly progressive motile sperm are used for insemination. Recently, a “rescue” version of this IVF method (termed “Rescue IVF in MBCD” in the present study) was reported [10] in which frozen-thawed mouse sperm were first washed by centrifugation and then capacitated in FERTIUP^®^ medium, while oocytes were pre-incubated in CARD^®^ medium, prior to co-incubating sperm and oocytes in FERTIUP^®^ medium for fertilization. However, it is unclear from this report what components were used in the FERTIUP^®^ and CARD^®^ media. Further, there was no direct comparison of fertilization rate of this “rescue” MBCD-GSH IVF version with the “standard” MBCD-GSH IVF version reported earlier [5–7,9], nor whether there was any correlation between sperm motility and fertilization rate to use as a guide for which of these two methods to use.

Therefore, in this study, we systematically investigated the correlation between sperm motility and fertilization rate, and compared the IVF efficiency of these two published IVF methods (MBCD-GSH IVF and Rescue IVF in MBCD) to that of two new methods (Rescue MBCD-GSH IVF and IVF in MBCD) devised in the present study using various sperm samples so as to establish general guidelines for mouse sperm cryorecovery by IVF.

## Materials and Methods

### Animals

Heterozygous male mice at 3 to 4 months of age for 113 unique knockout mouse lines on the inbred C57BL/6N genetic background were obtained from the KOMP Repository at the University of California at Davis (https://www.komp.org/faq.php). All mutant mouse lines were demonstrated to be fertile by natural mating or IVF using fresh sperm before sperm cryopreservation, and were used in different experiments. Individual number of mouse lines used in each experiment is provided in the Results below. Wild-type C57BL/6N mice (males at 3 to 4 months age and females at 3 to 4 weeks of age) were purchased from Charles River Laboratories (www.criver.com). All mice were housed in a specific pathogen free vivarium with a 14h on/10h off light cycle (7 am on and 9 pm off). Euthanasia was performed by CO_2_ asphyxiation followed by cervical dislocation according to the 2013 AVMA guidelines on euthanasia.

### Reagents and media

BD DifcoTM skim milk was purchased from Voigt Global Distribution Inc. (catalog # Difco-232100, Lawrence, KS), and equine chorionic gonadotropin (eCG) was purchased from the Lee BioSolutions (catalog # 493, St. Louis, Missouri). Human chorionic gonadotropin (hCG), D-(+)-raffinose pentahydrate, α-monothioglycerol (MTG), methyl-β-cyclodextrin (MBCD), reduced L-glutathione (GSH), bovine serum albumin (BSA, embryo tested), polyvinyl alcohol (PVA) and embryo tested water were purchased from Sigma-Aldrich Corp. (St. Louis, Missouri). M2 medium was purchased from LifeGlobal Group (catalog #ZFM2, http://www.ivfonline.com), and Research Vitro Fert (RVF) medium was purchased from Cook Medical, Inc. (Bloomington, Indiana). For IVF, the RVF medium was modified with addition CaCl_2_ to increase the Ca^2+^ concentration from 2.04 mM (regular concentration) to 5.14 mM (high concentration). MBCD medium (TYH medium containing 1 mg/mL PVA and 0.75 mM MBCD) [11] was prepared in-house. RVF and MBCD media as well paraffin oil (Fisher Scientific, Pittsburgh, Pennsylvania) were pre-equilibrated in CO_2_ incubators overnight or at least 1 hour prior to use.

### Sperm cryopreservation

CPA R18S3+MTG (18% raffinose, 3% skim milk and 477 µM α-monothioglycerol) was prepared using the method described previously [6]. Sperm from both cauda epididymides of sexually-mature males were collected in 0.5 mL or less R18S3+MTG. After 10 min of incubation at 37°C for dispersion, sperm were loaded into 1.8 mL cryovials (50 or 100 µL each, NUNC catalog #377267) and then cooled in LN2 vapor for 10 min before submersion and storage in liquid nitrogen.

### Sperm quality assessment

An IVOS II computer-assisted sperm analyzer (Hamilton Thorne, Beverly, Massachusetts) was used to assess sperm concentration and motility after the sperm suspension was diluted in M2 medium. Sperm total motility (% of sperm displaying any type of movement) and progressive motility (% of progressively swimming sperm with average path velocity ≥50 µm/s) were analyzed in chambers with depth 80 µm (2X-cel slides, Hamilton Thorne) by reading 8 to 10 fields (2000–3000 sperm) at 37°C.

### *In vitro* fertilization

Sperm were thawed in a 37°C water bath for 10 min, and cumulus-oocyte-complexes (COCs) were collected from superovulated C57BL/6N females 14–15 hours post hCG injection. Superovulation was induced by intraperitoneal (IP) injection of 5 IU eCG followed by IP injection of 5 IU hCG 47 hours later. IVF was performed in 5.5% CO_2_ in humidified air at 37°C. All medium drops were covered with paraffin oil. For paired or grouped IVF comparisons, the same drop volume of MBCD medium and GSH medium (RVF medium containing 1.0 mM GSH), the same amount and final concentration of sperm from the same sperm pool, about the same numbers of COC masses from the same pool of COCs, and the same time of sperm capacitation and COC treatment in GSH medium were used across treatments in each experiment. Four IVF methods were used and described below unless otherwise specifically stated in the results.

1. MBCD-GSH IVF (modification of a previously published method [5,6]): Briefly, thawed sperm were added directly into a pre-equilibrated 100 µL long-flat MBCD drop (about 0.5 × 1.5 cm size to increase the peripheral area for sperm collection after capacitation) if using 10 µL sperm; if using 40 to 50 µL sperm, sperm were distributed evenly into 3 pre-equilibrated 100 µL long-flat MBCD drops (12 to 15 µL sperm per MBCD drop). Sperm were incubated for 50–60 min before insemination. COCs collected from 5–20 superovulated females were distributed and incubated in 1 to 3 drops (200 or 250 µL each drop for COCs of 5–7 females) of RVF medium containing 1 mM GSH for 30 min before being inseminated with 20 to 40 µL sperm (final sperm concentration 1 to 5 × 10^5^ cells/mL), which were collected from the peripheral part of each MBCD drop. After 4 hours of co-culture, oocytes were washed through two 150 µL pre-equilibrated drops of RVF medium and incubated in two other 150-µL drops of RVF medium overnight until next day morning when the dishes were scored for IVF rate (2-cell rate).
2. Rescue MBCD-GSH IVF: After thaw, sperm were transferred into a microcentrifuge tube containing 1.2 mL RVF medium containing 239 µM MTG and centrifuged at 300 × g for 4 min. After removing the supernatant, sperm were resuspended in 100 µL (if using 10 µL sperm) or 300 µL (if using 40–100 µL sperm) pre-equilibrated MBCD medium, and then the sperm suspension was used to make 1 or 3 long-flat 100-µL drops (for 10 µL or 40–100 µL samples, respectively) covered with pre-equilibrated paraffin oil and incubated for 50–60 min before insemination. The other steps of IVF were the same as described above in the MBCD-GSH IVF method.
3. IVF in MBCD: COCs of 5–20 superovulated females were collected before thawing sperm and incubated in RVF medium containing 1 mM GSH for 50–70 min before insemination. After COC collection, sperm were thawed, and added into a 200 or 250 µL round MBCD medium drop if using 10 µL sperm; if using 40 to 50 µl sperm, sperm were distributed evenly into 3 pre-equilibrated 200 or 250 µL round MBCD drops (final sperm concentration 1 to 2 × 10^6^ cells/mL in the MBCD drops). After 30 min of sperm incubation, the pooled COCs were distributed evenly into the MBCD drop(s) containing sperm for fertilization (about 5–7 females/drop). After 4 h of co-culture, oocytes were washed and incubated in RVF medium as described above.
4. Rescue IVF in MBCD (modification of a previously published method [10]): Briefly, COCs were collected before thawing sperm and incubated in RVF medium containing GSH as described in the “IVF in MBCD” method above. After COC collection, sperm were thawed and washed in 1.2 mL RVF medium with 239 µM MTG by centrifugation as described in the Rescue MBCD-GSH IVF method above. The sperm were then resuspended in 200 or 250 µl (if using 10 µL sperm) or 600 or 750 µL (if using 40–100 µL sperm) pre-equilibrated MBCD medium (final sperm concentration 1 to 2 × 10^6^ cells/mL in MBCD medium), and this sperm suspension was used to prepare a 200 or 250-µL round drop or 3× 200 or 250-µL round drops (for 10 µL or 40–100 µL samples, respectively) covered with pre-equilibrated paraffin oil to be incubated for 30 min before COCs were distributed evenly into the MBCD drop(s) for fertilization (about 5–7 females/drop). After 4 h of co-culture, oocytes were washed and incubated in RVF medium as described above.

### Embryo transfer

For in vivo experiments, 2-cell stage embryos were transferred into the oviducts (10–13 for each oviduct, 20–25 per recipient) of 0.5 days post coitum pseudopregnant CD-1 recipient female mice anesthetized with 0.01 mL/g body weight of ketamine/xylazine solution (10 mg/mL ketamine and 1 mg/mL xylazine) by IP injection followed by subcutaneous injection of 0.1 mL of pain reliever Buprenex (0.03 mg/ mL; Western Medical Supply, Inc., Arcadia, CA, USA). Recipients were kept warm on a heating pad until fully recovered from anesthesia. All pregnant recipients were allowed to go to term and give birth. The health and number of pups born per litter were determined at the time of weaning at 21 days of age.

### Statistical analysis

GraphPad Prism 5 software (GraphPad Software, Inc., San Diego, California) was used for statistical analysis. Sperm fertilization rates, progressive sperm motilities and embryo transfer pup birth rates were arcsine transformed, and then group differences were detected by Student’s t-test or one-way ANOVA followed by Tukey HSD tests, and P<0.05 was considered significant. Data are expressed as mean (M) ± standard deviation (SD).

## Results

### Correlation between sperm motility and fertilization rate of MBCD-GSH IVF

**Figure 1:**
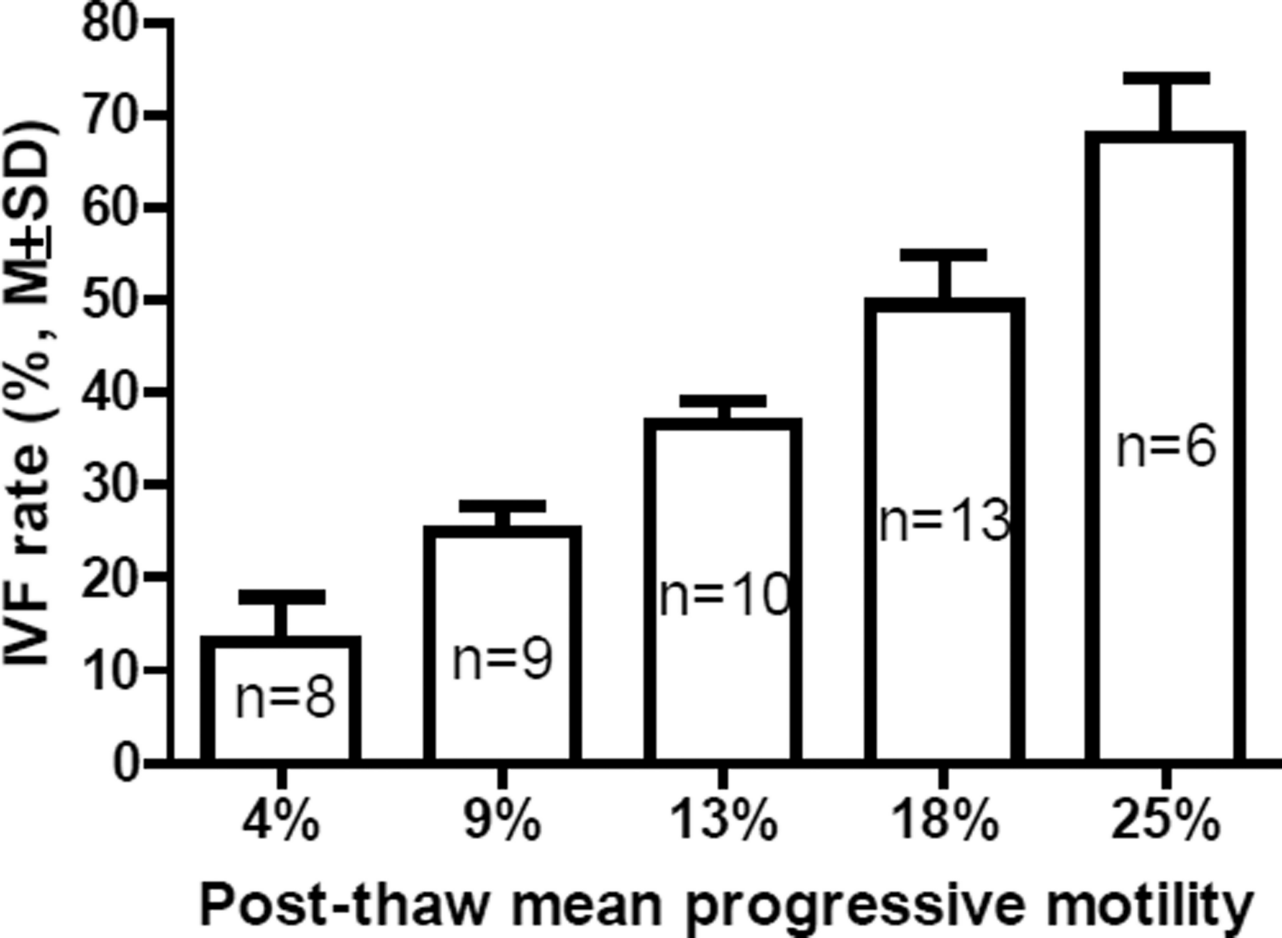
Correlation between post-thaw sperm progressive motility and fertilization rate of MBCD-GSH IVF, where n represents the number of IVF procedures performed for each mean progressive motility.

**Figure 2:**
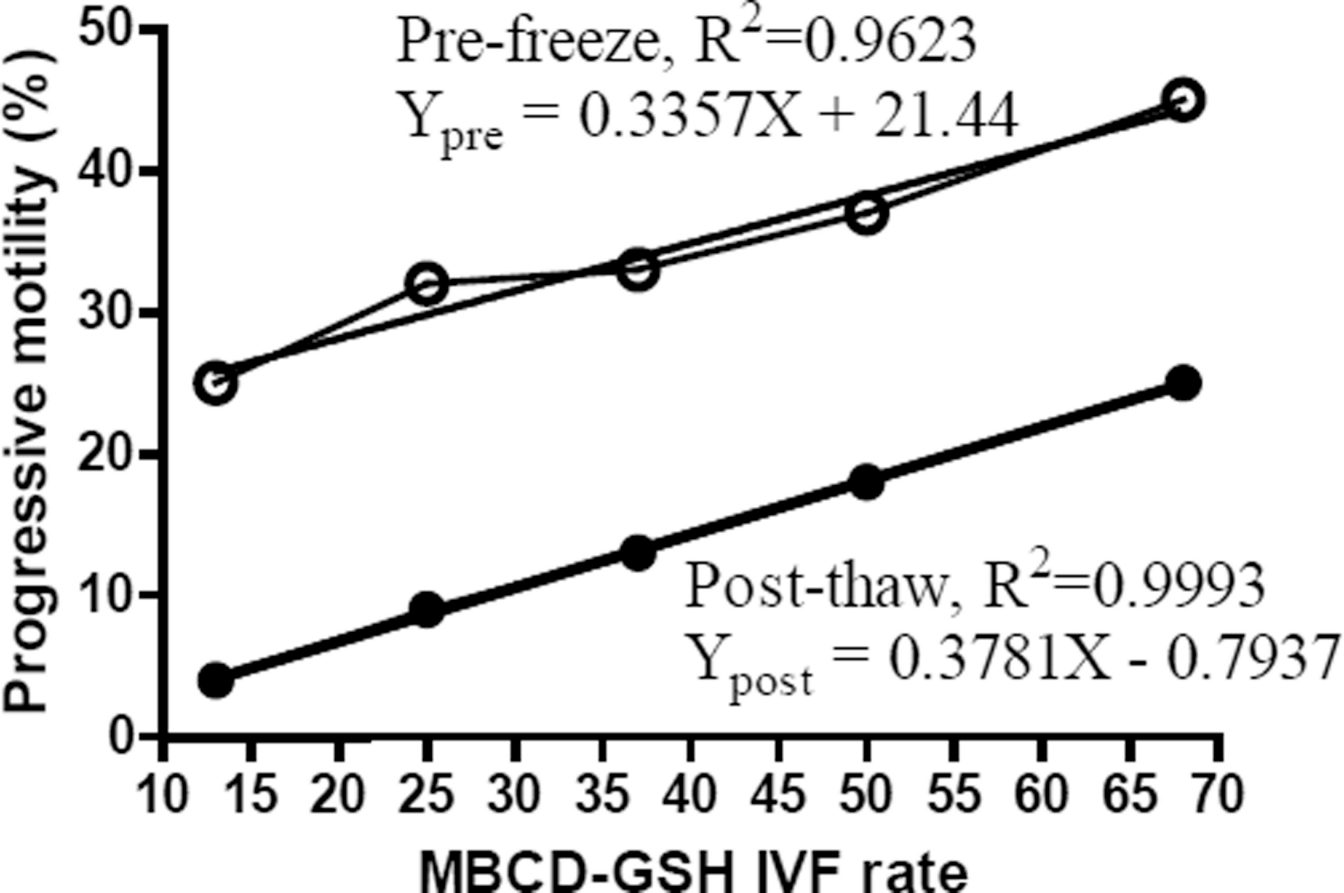
Linear regression between the mean of sperm progressive motility (pre-freeze and post-thaw) and the mean of fertilization rates of MBCD-GSH IVF. Y_pre_ = pre-freeze progressive motility; Y_post_ = post-thaw progressive motility; X = IVF rate.

Sperm samples of different mouse lines had different sperm motility. To determine the correlation between sperm motility and their fertilization abilities, MBCD-GSH IVF procedures were performed using cryopreserved sperm samples from 46 knockout mouse lines on a C57BL/6N genetic background. Although there was a large degree of variation in the IVF rate of thawed mutant sperm samples for each progressive motility (pre-freeze or post-thaw), there was a significant linear correlation between the mean of the IVF rate and the mean of the progressive motility after the fertilization rates of the 46 IVF procedures (1 IVF procedure per mouse line using sperm of a heterozygous male) were sorted and separated into 5 categories: 5–19%, 20–29%, 30–39%, 40–59%, and 60–80% (Figure 1). The R^2^ were 0.9623 and 0.9993 for pre-freeze and post-thaw progressive motility, respectively (Figure 2). The higher the mean of the progressive motility (especially the post-thaw progressive motility), the higher the mean of the IVF rate. IVF rates were usually less than 30% when post-thaw progressive sperm motility was less than 10%. The linear equations for the two variables (X, IVF rate; Y, pre-freeze or post-thaw progressive motility) containing slopes and Y-intercepts when X=0 are shown in Figure 2.

### Comparisons of cryorecovery IVF rates of different IVF methods

**Figure 3:**
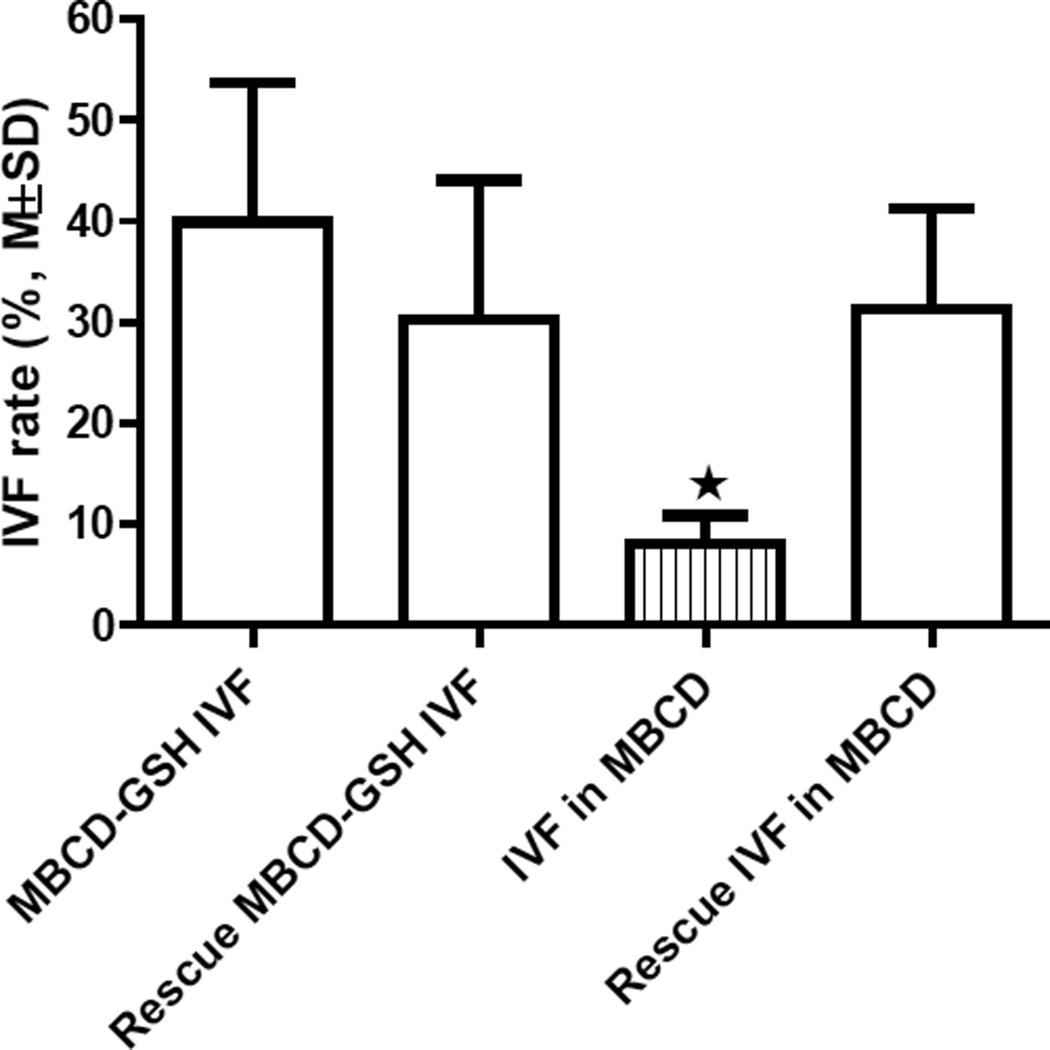
Comparisons of cryorecovery IVF rates of different IVF methods using wildtype C57BL/6N sperm. Each IVF procedure was repeated 3 times. Compared with the other 3 IVF methods, the IVF rate of the “IVF in MBCD” method was significantly lower (P<0.05).

The post-thaw sperm progressive motility of many cryopreserved mutant sperm samples on C57BL/6 backgrounds was less than 10%. To determine which IVF method should be used for a given sperm sample with a post-thaw progressive motility in the range of 5–10% and a sperm concentration of 30–50 × 10^6^/mL, we compared the IVF rates of 4 different IVF methods using 10 µL of C57BL/6N wildtype sperm with post-thaw progressive motility 5–8% from the same sperm pools (mixture of sperm from 3–5 males) for each IVF procedure. As shown in Figure 3, there was no significant difference in IVF rate among 3 of the IVF methods (MBCD-GSH IVF, Rescue MBCD-GSH IVF, and Rescue IVF in MBCD), but the IVF rate of the “IVF in MBCD” method was significantly lower than that of any of the other 3 IVF methods (P<0.05).

To determine if embryos derived by the 4 different IVF methods have the same developmental potential, IVF procedures using different methods and cryopreserved sperm from total 59 knockout mouse lines on C57BL/6N background were transferred into the oviducts of pseudopregnant foster mothers. Results summarized in Table 1 show that there is no significant difference in the pup birth rate of embryos derived from the 4 IVF methods (P>0.05), indicating that 2-cell embryos produced by all 4 of the IVF methods developed normally.

### Effect of CPA on IVF rate

**Figure 4:**
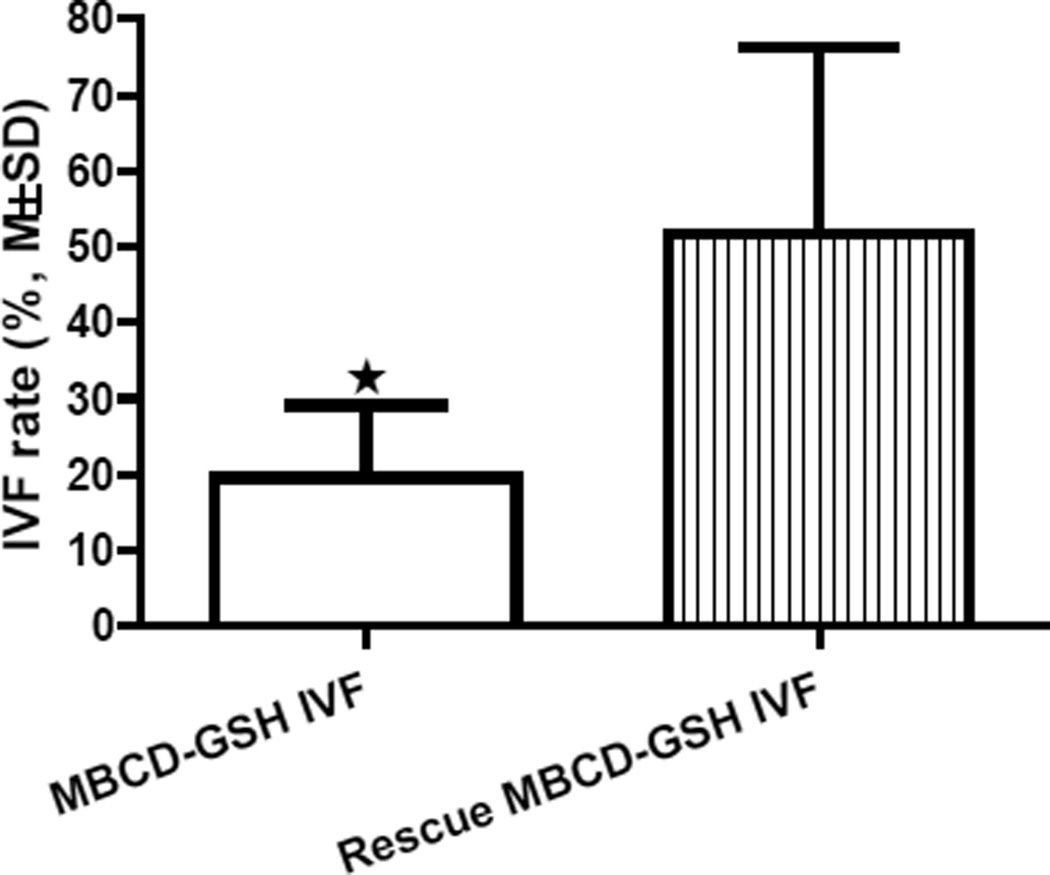
Effect of high CPA concentration on IVF rates of MBCD-GSH IVF and Rescue MBCD-GSH methods (n=4, P<0.05).

To determine if a high concentration of CPA inhibits sperm capacitation and fertilization, MBCD-GSH IVF and Rescue MBCD-GSH IVF methods were compared in pairs using the same amount of cryopreserved sperm from the same males of 4 knockout mouse lines. For each MBCD-GSH IVF procedure, a high volume (20 µL) of thawed sperm in CPA was mixed with 100 µL of MBCD medium (final concentration raffinose 3% and skim milk 0.5%, w/v), and for each Rescue MBCD-GSH IVF, 20 µL of thawed sperm were washed by centrifugation to remove CPA and then resuspended in 120 µL of MBCD medium for capacitation. The results summarized in Figure 4 show that the IVF rate of the Rescue MBCD-GSH IVF was significantly higher (P<0.05) than that of the MBCD-GSH IVF, indicating that the presence of a high concentration of CPA (3% raffinose and 0.5% skim milk) in the MBCD medium significantly inhibits the ability of MBCD to enhance sperm capacitation.

### Effect of immotile sperm on IVF rate

**Figure 5:**
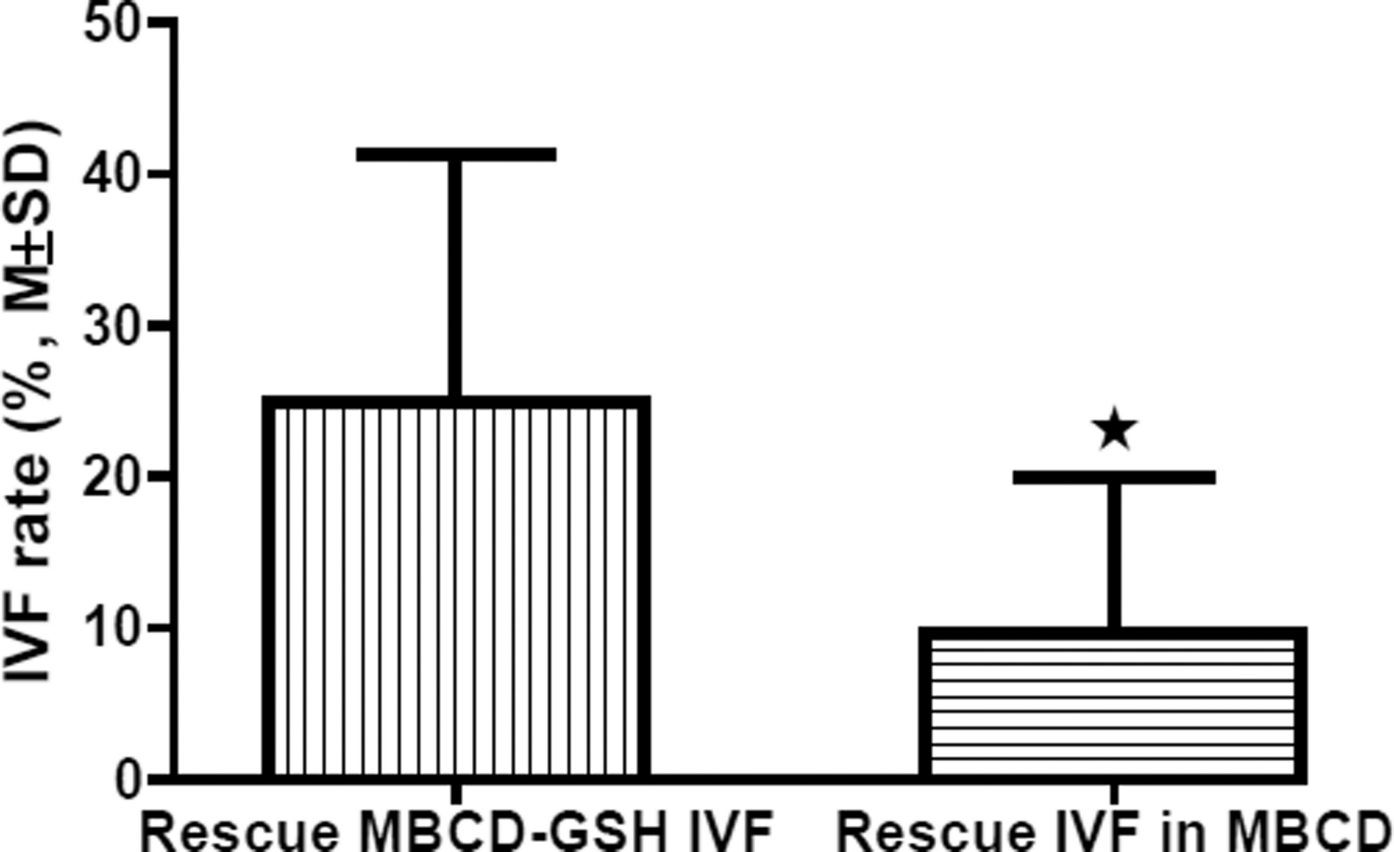
Comparison of IVF rates of Rescue MBCD-GSH IVF and Rescue IVF in MBCD using sperm samples with high concentration and low motility (n=4, P<0.05).

We also compared the fertilization rates of the Rescue MBCD-GSH IVF and Rescue IVF in MBCD methods using sperm samples from 4 knockout mouse lines with high sperm concentration but low post-thaw motility (total motility 9–25% and progressive motility 5–9%) to determine if immotile sperm affect sperm capacitation and fertilization. The final sperm concentration was 5–8 × 10^6^ cells/mL in the 100-µL MBCD drop (sperm capacitation drop) for the Rescue MBCD-GSH IVF method and 2.5–4 × 10^6^ cells/mL in the 200-µL MBCD drop (for both sperm capacitation and fertilization) for the Rescue IVF in MBCD method. The results summarized in Figure 5 show that the IVF rate of the Rescue MBCD-GSH IVF, in which sperm were pre-incubated in the MBCD medium drop and only motile sperm with minimal immotile sperm were used for insemination in the fertilization drop (GSH medium drop), was significantly higher than that of the Rescue IVF in MBCD, in which all sperm (dead, immotile alive, and motile) were present in the fertilization drop (MBCD medium drop). These results indicate that the presence of a high number of immotile sperm significantly inhibits the fertilization after capacitation in MBCD medium, but does not significantly affect sperm capacitation in MBCD medium.

### Effect of MTG on protecting sperm fertilization rate during centrifugation

**Figure 6:**
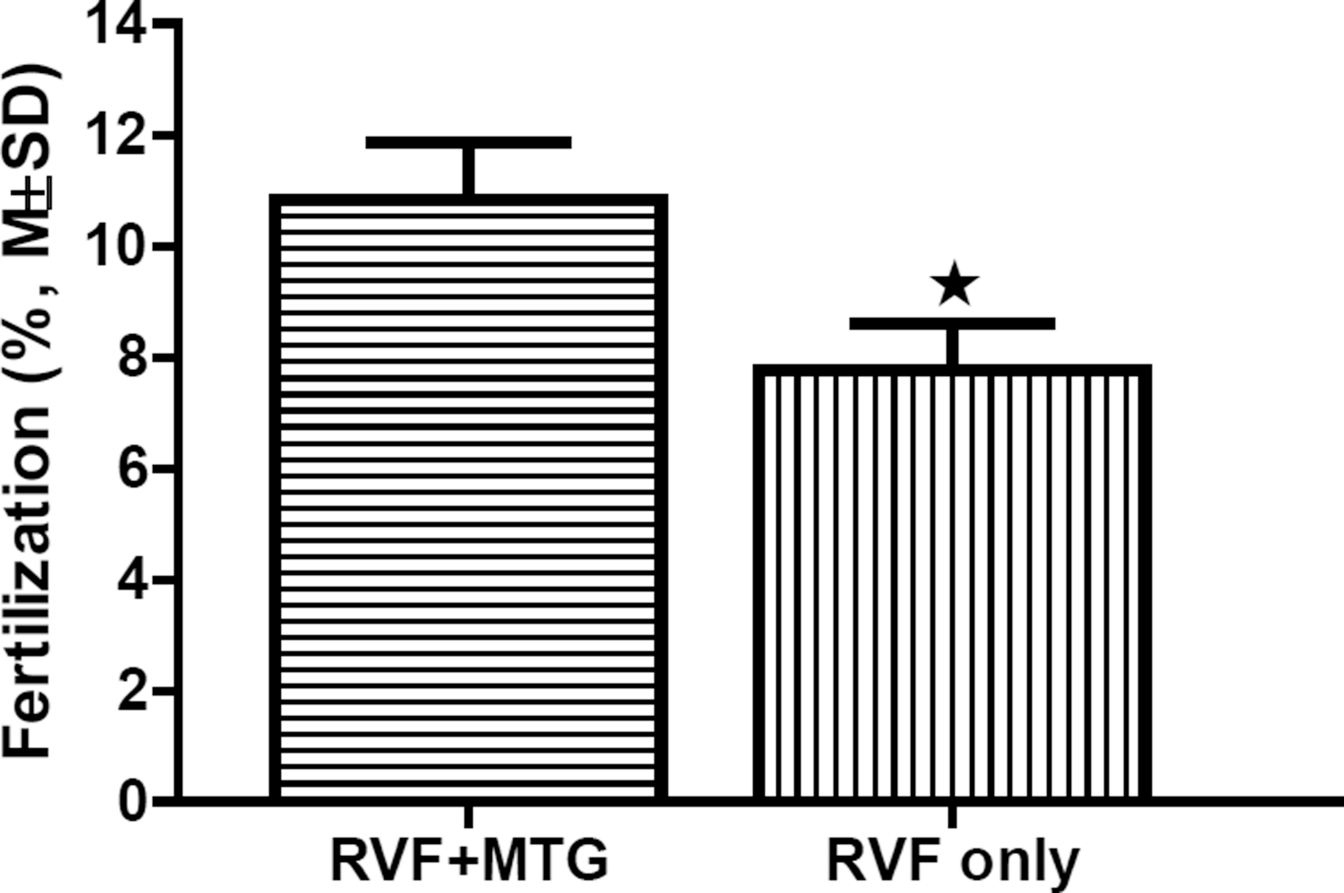
Comparison of IVF rates of Rescue IVF in MBCD using sperm centrifuged in the presence (RVF+MTG) or absence of MTG (RVF only). n=3, P<0.05.

Centrifugation is a step of the Rescue MBCD-GSH IVF and Rescue IVF in MBCD methods necessary for the removal of cryoprotectants and adjustment of sperm concentration. However, mouse sperm are sensitive to mechanical stress induced by centrifugal force, and during centrifugation sperm can undergo oxidative stress. To determine if the addition of MTG to a frozen-thawed sperm suspension protects sperm fertility during centrifugation, the fertilization rates of Rescue IVF in MBCD procedures were compared after sperm were centrifuged at 300×g for 3, 4, or 5 min in 1.2 mL RVF medium in the presence or absence of 239 µM MTG. To readily determine the effect of MTG, a low sperm concentration (final 1 × 10^5^ cells/mL) with low post-thaw progressive motility (3%) taken from the same pool of cryopreserved C57BL/6N sperm (mixture of sperm from 3 males) was used in each of the 3 paired IVF experiments. The IVF rates obtained in the presence of MTG for all 3 centrifugation times (3, 4, and 5 min) were higher than that for sperm centrifuged for the same times in the absence of MTG. Data analysis shows that this difference is significant (P<0.05, Figure 6), which indicates that MTG can protect sperm fertilization ability during post-thaw centrifugation.

## Discussion

Sperm parameters, including sperm concentration, progressive motility, and morphology, are important markers of sperm quality. Progressive motility is characterized by high velocities and symmetrical, low-amplitude flagella bends [12]. To our knowledge, this is the first report studying the correlation between sperm progressive motility and fertilization rate using the MBCD-GSH IVF method, which is the current gold-standard method for mouse sperm cryorecovery [5,6,9,13,14]. We found that both the pre-freeze and especially post-thaw sperm progressive motilities are highly predictive of sperm fertilizing rate and MBCD-GSH IVF outcome, which has great economic importance for cryopreservation and cryorecovery of genetically modified mouse lines. Using the linear equations obtained from this study, pre-freeze and (especially) post-thaw progressive motility can be used to predict the IVF rate of a sperm sample, as long as the genetic modification itself is not a mitigating factor. Therefore, for a given sperm sample the number of female mice to be superovulated, the number of IVF media and IVF dishes to be prepared and the number of sperm samples to be thawed can be known in advance according to a specific embryo production goal. It is important to cryopreserve sperm with better progressive motility when possible and to assess sperm quality and record sperm parameters when banking mouse sperm.

So far, two IVF methods, MBCD-GSH IVF [5–7,13,14] and Rescue IVF in MBCD [10], for cryorecovery of mouse sperm using MBCD and GSH have been reported. MBCD-GSH IVF involves pre-incubation of sperm in an MBCD medium drop to enhance sperm capacitation, followed by collection of capacitated sperm from the MBCD drop for fertilizing COCs that are incubated in a GSH-containing medium drop. The advantages of this method include removal of immotile sperm and CPA from the subsequent fertilization process and centrifugation is not needed, but CPA is present in sperm capacitation medium and not all capacitated sperm can be collected for insemination. Therefore, MBCD-GSH IVF method is not good for sperm samples with small volume, low sperm concentration or low sperm motility (Table 2). The Rescue IVF in MBCD method overcomes some disadvantages of the MBCD-GSH IVF method by removal of CPA from sperm using centrifugation and addition of COCs to the MBCD medium drop after pre-incubation in medium containing GSH for fertilization, but all sperm including immotile sperm are present in the sperm-COC co-incubation drop.

To address the deficiencies of these previously published methods, we developed two new IVF methods, Rescue MBCD-GSH IVF and IVF in MBCD methods, and compared their IVF efficiency with that of the two extant methods. Unlike in MBCD-GSH IVF, sperm recovered using our Rescue MBCD-GSH IVF method are washed by centrifugation before incubation in MBCD medium. Our results show that the IVF rates of these two methods are not significantly different until ≥20 µL of sperm in CPA is added to a 100 µL MBCD drop, and then the fertilization rate of the Rescue MBCD-GSH IVF is significantly higher than that of the MBCD-GSH IVF. This observation is most likely due to increasing amounts of CPA that inhibits the ability of MBCD to enhance sperm capacitation. It has been reported that casein, a major component of skim milk used in CPA (R18S3), inhibits the loss of cholesterol from bull sperm membranes and, therefore, inhibits sperm capacitation [15]. These observations indicate that, when performing MBCD-GSH IVF using a large volume (for example 100 µl in a cryovial) of low-concentration sperm, the CPA should be removed using the Rescue MBCD-GSH IVF method so as to improve fertilization.

IVF in MBCD and Rescue IVF in MBCD both methods involve co-incubation of sperm and oocytes in MBCD medium after sperm pre-incubation in the same MBCD medium drop, and COC pre-incubation in GSH-containing medium. The difference between these two IVF methods is that, in the rescue method, sperm are washed by centrifugation prior to incubation in the MBCD drop. Of the 4 IVF methods tested in this report, the fertilization rate of the IVF in MBCD method was significantly lower than that of any of the other 3 IVF methods. Since the presence of low-concentration CPA in the MBCD drop in the MBCD-GSH IVF method did not inhibit sperm capacitation or fertilization, the cause of the low fertilization rate of the IVF in MBCD method is most likely due to the presence of CPA in the fertilization drop, in which the sperm and oocytes were co-incubated. This observation indicates that the fertilization process is more sensitive to CPA than the sperm capacitation process. The significantly higher IVF rate of the Rescue IVF in MBCD method, in which the sperm were washed by centrifugation to remove CPA, further confirms this. The rescue IVF in MBCD method is especially good for cryorecovery of sperm samples with small volumes (for example cryostraws containing 10 µL sperm each) of low-concentration sperm because there is no need to collect motile sperm from the MBCD medium (as is done in the MBCD-GSH IVF and Rescue MBCD-GSH IVF methods), and, therefore, all of the capacitated sperm are co-incubated with COCs for fertilization, and no sperm are wasted.

High concentrations of immotile and dead sperm have been reported to inhibit fertilization [16,17]. This is probably due to the fact that dead and injured sperm produce excessive reactive oxygen species (ROS) [18] and other substances that block the fertilization process [19]; this hypothesis is supported by reports demonstrating improved fertilization following the separation of viable from non-viable sperm prior to IVF [7,17]. In the present study, we used sperm samples with high sperm concentration and low motility to compare the fertilization rates of the Rescue MBCD-GSH IVF and Rescue IVF in MBCD methods after removal of CPA by centrifugation. We found that the fertilization rate of the Rescue IVF in MBCD method was significantly lower than that of the Rescue MBCD-GSH IVF method, confirming that the presence of a large number of immotile sperm in the sperm-oocyte co-incubation drop is detrimental to fertilization.

Sperm are particularly susceptible to oxidative stress by virtue of their high unsaturated fatty acid content and limited availability of intracellular antioxidant enzymes [20]. ROS are produced by sperm undergoing cooling, freezing, and thawing [21–25], and overproduction of ROS during cryopreservation could damage sperm, impairing their fertilizing ability and the subsequent embryo development. Antioxidant supplementation, for the purposes of inhibition of lipid peroxidation and maintenance of sperm motility and fertilization ability, has been documented in many mammalian species and humans [6,22–25]. In this report, we demonstrated that supplementation of sperm washing medium with MTG, an antioxidant, during centrifugation preserves sperm fertilization ability.

In conclusion, the present study investigated the correlation between sperm motility and fertilization rate of frozen-thawed mouse sperm on C57BL/6 background, and compared the efficiency of different chemical-assisted IVF methods using various sperm samples, and found 1) a significant linear correlation between sperm progressive motility and fertilization rate, 2) that a high concentration of CPA impairs both sperm capacitation and fertilization processes, and 3) that the presence of a large number of immotile sperm in the sperm-oocyte co-incubation drop is detrimental to fertilization. Based on these comparisons (Table 2), we found that sperm pre-incubated in MBCD medium followed by fertilization in medium containing GSH (the MBCD-GSH IVF method) can separate motile from immotile sperm, and that sperm washing by centrifugation can remove CPA (the Rescue MBCD-GSH IVF method); both of these processes facilitate sperm capacitation and fertilization. However, sperm samples with small volume and low sperm count should be centrifuged first and then incubated in MBCD prior to IVF (the Rescue IVF in MBCD method). Supplementing sperm washing medium with MTG preserves post-thaw sperm fertilization ability during centrifugation.

## Figures and Tables

**Table 1:** Comparisons of pup birth rates of embryos derived by different IVF methods, n= number of knockout mouse lines = number of IVF/ET procedures, P>0.05.

IVF method	n	Total No. of 2-cell Embryos Transferred	Total No. of Pups Born	Pup Birth Rate (%, M ± SD)
**MBCD-GSH IVF**	18	792	309	38.5 ± 9.4
**Rescue MBCD-GSH IVF**	14	616	252	40.7 ± 9.2
**IVF in MBCD**	12	466	152	33.6 ± 8.3
**Rescue IVF in MBCD**	15	696	254	36.6 ± 10.4

Note: Sperm of a total 59 knockout males on C56BL/6N genetic background and oocytes of wild-type C57BL/6N females were used for the IVF procedures. The mutant mouse lines in each of the 4 groups were not the same.

**Table 2:** Comparisons of the four IVF methods.

	MBCD-GSH IVF	Rescue MBCD-GSH IVF	IVF in MBCD	Rescue IVF in MBCD
**Fertilization**	In GSH medium	In GSH medium	In MBCD medium	In MBCD medium
**Centrifugation**	No	Yes	No	Yes
**Advantages**	No sperm centrifugation stress; less immotile sperm and CPA present at fertilization	CPA removed; less immotile sperm present at fertilization	No sperm centrifugation stress; all sperm available for insemination	All sperm available for insemination; CPA removed
**Disadvantages**	CPA present in capacitation medium; some motile sperm wasted	Centrifugation stress; some motile sperm wasted	Both CPA and immotile sperm present at both capacitation and fertilization steps	Centrifugation stress; immotile sperm present at fertilization step

## Abbreviations

CPA: Cryoprotective Agent
COCs: Cumulus-Oocyte-Complexes
eCG: Equine Chorionic Gonadotropin
Glu: Glutamine
GSH: Reduced Glutathione
hCG: Human Chorionic Gonadotropin
MBCD: Methyl-β-Cyclodextrin
MTG: Monothioglycerol
PVA: Polyvinyl Alcohol
R18S3: 18% Raffinose and 3% Skim Milk
